# Modulatory Effects of *Echinococcus multilocularis* emu-let-7-5p on the Immunological Functions of RAW264.7 Macrophages

**DOI:** 10.3389/fvets.2021.663497

**Published:** 2021-04-15

**Authors:** Xiaoliang Jin, Yating Li, Xing Yang, Yadong Zheng

**Affiliations:** ^1^Department of Ophthalmology, Shanghai Ninth People's Hospital, Shanghai Jiaotong University School of Medicine, Shanghai, China; ^2^State Key Laboratory of Veterinary Etiological Biology, Key Laboratory of Veterinary Parasitology of Gansu Province, Lanzhou Veterinary Research Institute, Chinese Academy of Agricultural Sciences (CAAS), Lanzhou, China; ^3^Department of Medical Microbiology and Immunology, School of Basic Medicine, Dali University, Dali, China; ^4^Jiangsu Co-innovation Center for Prevention and Control of Important Animal Infectious Diseases and Zoonoses, Yangzhou, China

**Keywords:** *Echinococcus multilocularis*, emu-let-7-5p, pathogen-host interplay, cytokine, LPS/TLR4 signaling pathway

## Abstract

*Echinococcus multilocularis* is a zoonotic tapeworm with great medical significance. In *E. multilocularis*-infected mice, parasite-derived let-7-5p (emu-let-7-5p) is present in the sera, but its role remains unclear. Using qPCR, ELISA and flow cytometry, the immunomodulatory effects of emu-let-7-5p were *in vitro* investigated using RAW264.7 macrophages. Compared with the control, emu-let-7-5p significantly downregulated IL-1α (*p* < 0.05), but anti-inflammatory cytokine genes remained to be stably expressed in the treated macrophages. Moreover, significantly decreased expression of *ripk1* and *nf-kB*, key components in the LPS/TLR4 signaling pathway, was also observed in the emu-let-7-5p-transfected cells (*p* < 0.05). Furthermore, CD40 was upregulated in these transfected cells (*p* < 0.05), while CD86, CD54 and CD80 remained unchanged compared that in the control. These results demonstrate a property of emu-let-7-5p in regulation of immune functions of macrophages, making it be possibly involved in the pathogen-host interplay during *E. multilocularis* infection.

## Introduction

*Echinococcus multilocularis* is a causative pathogen of alveolar echinococcosis in human beings, which is listed as one of neglected tropical diseases by WHO (https://www.who.int/teams/control-of-neglected-tropical-diseases). Naturally, this parasite needs two different hosts to complete its life cycle, and it is mainly transmitted between foxes as a definitive host and wild rodents as an intermediate host. Occasionally, human beings are infected when consuming egg-contaminated water or food, and activated eggs develop into metacestodes in the liver or lung (1). If not properly treated or untreated, the disease is lethal due to the “invasive” growth of a parasite. Clinically, alveolar echinococcosis has a long latent period of up to 15 years, at least partially, due to the ability of parasites in modulation of immune responses (1). During *E. multilocularis* infection, the immune functions of immune cells are dynamically modified, including cytokine and NO dysregulation (2).

microRNAs (miRNAs) are one of small regulatory RNA species, which normally interact with the binding site(s) in the 3′ untranslated region and induce translational repression or degradation of targeted mRNA. With a few exceptions, all the parasites investigated so far, including *Echinococcus* species, encode miRNAs, some of which have been found to be closely associated with their infections (3). Among them, let-7 is conserved across Bilateria and has complex evolutionary patterns (4). Previous studies showed that let-7 was potentially involved in the development and infection of parasites (5–7). For instance, infection of *Cryptosporidium parvum* induced let-7i downregulation in infected cholangiocytes, thus giving rise to upregulation of its target Toll-like receptor 4. It was further shown that low expression of let-7i led to a remarkable decrease of parasite burden, suggesting a role in immune responses against *C. parvum* infection (6). During *Trichuris suis* infection, parasite let-7 was found to be present and significantly upregulated in the sera of infected pigs compared with that of the controls. This increased let-7 was hypothesized to be involved in the regulation of IL-13 translation (7). In cestodes, canonical mature let-7, let-7-5p, was found to be one of highly-expressed miRNAs (8–10). Moreover, let-7-5p was recently shown to be present in the sera of *E. multilocularis*-infected mice (11) and in extracellular vesicles released by *Taenia crassiceps* (12). In *E. multilocularis* infection, the potential primary source of the serological let-7-5p is from parasite-released extracellular vesicles, which have been verified to encapsulate this miRNA in multiple parasites (12–14). These let-7-5p-containing extracellular vesicles are easily taken up by host cells, primarily by macrophages (14). These results suggest that these parasites secrete let-7-5p into host tissues during infection, but its role remains unknown.

Using RAW264.7 macrophages, the effects of *E. multilocularis* let-7-5p (emu-let-7-5p) on nitric oxide (NO) production, and expression of cytokines, costimulatory molecules and key components in the LPS/TLR4 signaling pathway were assessed. The results demonstrated a modulatory property of emu-let-7-5p, which is possibly involved in the pathogenesis.

## Materials and Methods

### Ethics Statement

All the experiments were assessed and approved by Ethics Committee of Lanzhou Veterinary Research Institute, Chinese Academy of Agricultural Sciences. Animal experiments were performed strictly according to the guidelines.

### Cells and Transfection

RPMI-1640 supplemented with 10% fetal bovine serum (Invitrogen) was used to culture RAW264.7 macrophages at 37°C, 5% CO_2_. When cells reached at 70–80% confluence, they were digested and the viability was determined using trypan-blue (Sigma), followed by cell seeding into 6-well plates (Costar) with a total of 1.0 × 10^6^ cells per well. Prior to transfection, cells were incubated until the confluence was around 70–80%. emu-let-7-5p mimics (catalog number: 4464066, Invitrogen) transfection and cell stimulation were conducted as previously reported (15). For transfection, the mimics were added at a final concentration of 30 nM per well. Then cells and culture supernatant were collected 24 h post stimulation with LPS and IFN-γ (Sigma) at a final concentration of 100 and 10 ng/mL, respectively. As a control, negative control RNA (catalog number: 4464058, Invitrogen) was used.

### Total RNA Extraction and Reverse Transcription

Cells were first washed several times in ice-cold PBS prepared using DEPC-treated water. RNA extraction was performed using TRIzol (Invitrogen) according to the instructions. In short, samples were thoroughly homogenized and then mixed with chloroform, followed by RNA precipitation. After wash, the pellets were resuspended in DEPC-treated water. RNA concentration and quality were analyzed using Nanodrop (ThermoFisher Scientific) and formaldehyde-denatured agarose gel electrophoresis, respectively.

Using oligo (dT)_18_, 1.5 μg of total RNA were reversely transcribed using RevertAid First Strand cDNA Synthesis Kit (ThermoFisher Scientific) according to the instructions. The products were immediately diluted by addition of 130 μL nuclease-free water and used for qPCR analysis of protein-encoding genes.

### NO Determination

NO levels in the culture supernatant were measured using Griess reagent (Invitrogen) as previously described (15). Shortly, the culture supernatant was thoroughly mixed with freshely-prepared Griess reagent and distilled water, followed by incubation in dark. Then the absorbance at 570 nm was measured by a microplate reader (Bio-Rad). Every sample was tested in triplicate and the final NO levels were calculated from three independent experiments.

### qPCR Analysis

The relative expression levels of 19 protein-coding genes were analyzed by qPCR, including *inos*, 7 cytokine genes (*il-4, il-10, tnf-*α, *il-1*α, *il-1*β, *il-6*, and *il-12B*) and 11 key genes in the LPS/TLR4 pathway (*cd14, tlr4, myd88, tirap, ticam1, ticam2, irf-3, irf-5, ripk1, nf-kB*, and *ap-1*). qPCR was performed using All-in-One qPCR Mix (GeneCopoeia) as previously described (15). All the primers for protein-encoding genes were commercially available from GeneCopoeia and β*2m* was used as an internal reference gene. For emu-let-7-5p qPCR, a poly-A reverse transcription-PCR strategy was adopted as previously described (12). Briefly, 2 μg of total RNA were polyadenylated and synthesized into cDNA using All-in-One miRNA First-Strand cDNA Synthesis Kit (GeneCopoeia) with a primer 5′- GCGAGCACAGAATTAATACGACTCACTATAGG(T)_12_VN-3′ (V = A, G, C; N = A, T, G, C). Then diluted cDNA was used for qPCR reactions with primers em-let7F: 5′- TGGGCTGAGGTAGTGTTTCG-3′ (forward) and universal R: 5′- GCGAGCACAGAATTAATACGAC-3′ (reverse).

In all qPCR reactions, every primer was at a final concentration of 200 nM. qPCR was performed using an ABI7500 thermocycler (ThermoFisher Scientific) with the following steps: 95°C for 10 min, followed by 40 cycles of 95°C for 10 s and 60°C for 1 min. The reactions without reverse transcription or cDNA template were included as negative controls. The expression levels of individual target genes were determined by 2^−ΔΔCt^ formula. Every sample was tested in triplicate and the final gene expression levels were calculated from three independent experiments.

### Sandwich Enzyme-Linked Immunosorbent Assay

The levels of IL-1α and IL-6 in the culture supernatant were determined by sandwich enzyme-linked immunosorbent assay (sELISA) using DuoSet IL-1α and IL-6 Kits (R&D Systems) as previously described (15), respectively. In short, diluted capture antibody was added into 96-well plates (Costar), followed by blocking with Reagent Diluent. After addition of samples or standards, the plates were successively incubated with Detection Antibody and Streptavidin-HRP. After addition of Stop Solution, the absorbance was determined by subtracting the values at 540 or 570 nm from the values at 450 nm. Every sample was set in triplicate.

### Flow Cytometry

The expression of cell surface costimulatory molecules of macrophages, including CD40, CD54, CD80, and CD86, was quantified by flow cytometry. Cells were prepared and transfected as described above. Twenty-four hours after transfection, cells were harvested and washed three times in ice-cold PBS. Then, cells were resuspended in the Binding buffer (BD Biosciences), followed by incubation with FITC anti-CD40 (1 μg/10^6^ cells, BioLegend), APC anti-CD54 (0.06 μg/10^6^ cells, BioLegend), or PE anti-CD80/86 (0.5 μg/10^6^ cells, BioLegend) for 30 min at 4°C in the dark. Cells were then washed in ice-cold PBS, resuspended in 500 μL of the Binding buffer (BD Biosciences) and analyzed using a flow cytometer (Merk). The data were analyzed using Guava (3.1.1), and the protein expression levels were expressed as mean fluorescence intensity (MFI). The final results were derived from three independent experiments.

### Statistical Analysis

The difference between two groups was calculated using a two-tailed unpaired *t*-test (GraphPad Prism 5). If a *p*-value is < 0.5, the difference is considered to be significant.

## Results and Discussion

**Figure 1:**
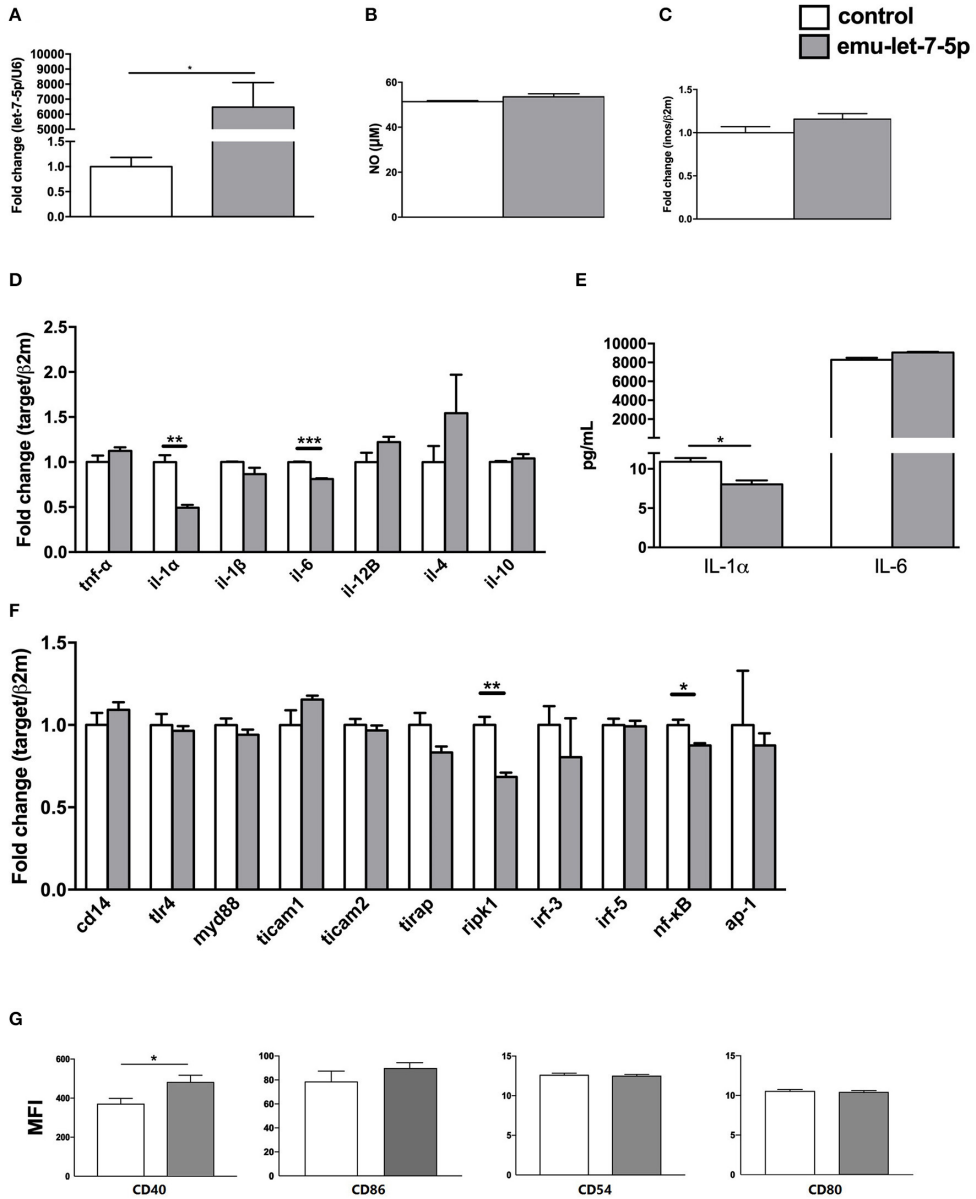
Immunomodulatory effects of emu-let-7-5p in the transfected RAW264.7 macrophages. **(A)** qPCR analysis of the levels of emu-let-7-5p in mimics-transfected cells. Data are expressed as mean ± s.e.m; ^*^p < 0.05. Data for the final analysis are from three independent experiments. **(B)** NO secretion post transfection with emu-let-7-5p. Data are expressed as mean ± s.e.m. Data for the final analysis are from three independent experiments. **(C)** qPCR analysis of *inos* expression post transfection with emu-let-7-5p. Data are expressed as mean ± s.e.m. Data for the final analysis are from three independent experiments. **(D)** qPCR analysis of the expression of pro- and anti-inflammatory cytokine genes post transfection with emu-let-7-5p. Data are expressed as mean ± s.e.m; ^**^p < 0.01, *** *p* < 0.001. Data for the final analysis are from three independent experiments. **(E)** Determination of IL-1α and IL-6 levels by ELISA. Data are expressed as mean ± s.e.m; *p < 0.05. Data for the final analysis are from three independent experiments. **(F)** qPCR analysis of the expression of key components in the LPS/TLR4 signaling pathway post transfection with emu-let-7-5p. Data are expressed as mean ± s.e.m; *p < 0.05, **p < 0.01. Data for the final analysis are from three independent experiments. **(G)** Flow cytometry of the expression of CD40, CD86, CD54 and CD80 post transfection with emu-let-7-5p. Data are expressed as mean ± s.e.m; *p < 0.05. Data for the final analysis are from three independent experiments. MFI, mean fluorescence intensity.

As expected, the level of emu-let-7-5p was significantly increased in the mimics-transfected group compared with the control (*p* < 0.05, Figure 1A). It was shown that emu-let-7-5p did not alter NO levels in the transfected macrophages after stimulation with LPS and IFN-γ (*p* > 0.05, Figure 1B). In accordance with this result, the expression of *inos*, a gene responsible for NO production, almost kept constant in both emu-let-7-5p- and control-transfected cells (*p* > 0.05, Figure 1C). It is well known that, as a reactive nitrogen intermediate, NO plays a role in *E. multilocularis* infection via modulating parasitic microenvironment (2). A number of parasite-origin molecules, such as miR-71 (15), 14-3-3 (16) and TegP11 (17), have been found to be capable of impairing NO secretion by macrophages. Taken together these results suggest that emu-let-7-5p has no role in modulating NO production during *E. multilocularis* infection.

To assess the effects of emu-let-7-5p on cytokine expression, seven cytokine genes were selected, including two anti-inflammatory cytokine genes *il-4* and *il-10*, and five pro-inflammatory cytokine genes *tnf-*α, *il-1*α, *il-1*β, *il-6*, and *il-12B* that are essential for parasite clearance and parasitism in echinococcosis (18, 19). As shown in Figure 1D, although all the cytokine genes showed a fluctuation in expression, only *il-1*α and *il-6* were significantly downregulated in the emu-let-7-5p-transfected macrophages (*p* < 0.01) by 0.49- and 0.81-fold compared with the control, respectively. The ELISA results further confirmed that only IL-1α was significantly decreased (*p* < 0.05, Figure 1E). *E. multilocularis* infection is characterized by a shift from a Th1 cytokine response at the early stage to a Th2 cytokine response at the late chronic stage, and Th1 cytokines including IL-1 and IL-12 contribute to parasite clearance, while Th2 cytokines including IL-4 and IL-13 promote parasitism (19). Consistently, IL-1α and IL-6 were highly expressed during the first 129 days of infection and then downregulated afterwards in a murine secondary hydatidosis model (18). In future experiments, it is interesting to determine the level of emu-let-7-5p in sera during different infectious periods and then to further analyze its contribution to IL-1α downregulation in *E. multilocularis*-infected animals.

It has already been found that some parasite molecules are capable of modulating the expression of pro- and/or anti-inflammatory cytokines (2). A recent study also demonstrated that extracellular vesicles released by *E. multilocularis* induced downregulation of *il-1*α and *il-1*β (20). Extracellular vesicles are membrane-enveloped vehicles in a nano-size and carry a plethora of active molecules such as proteins and miRNAs, being involved in parasitic infections (21). Consistent with the finding that let-7-5p was present in the sera of infected mice (11), it existed in the extracellular vesicles secreted by *E. multilocularis* (22). Therefore, emu-let-7-5p transported by extracellular vesicles may be beneficial for *E. multilocularis* parasitism via repression of pro-inflammatory cytokine IL-1.

For evaluation of the effects of emu-let-7-5p on the LPS/TLR-4 signaling pathway, 11 key genes *cd14, tlr4, myd88, tirap, ticam1, ticam2, irf-3, irf-5, ripk1, nf-kB*, and *ap-1* were included. In the transfected cells, emu-let-7-5p was shown to significantly downregulate *ripk1* and *nf-kB* (*p* < 0.05), with the remaining genes being stably expressed compared with these in the control (Figure 1F). But it is still not clear whether *ripk1* and *nf-kB* suppression is related to the downregulation of *il-1*α and *il-6* or not (Figure 1D). RIPK1, a regulator of inflammation, apoptosis and necroptosis, is an enzyme with multiple functions which plays a different role in distinct tissues or organs. RIPK1 can suppress apoptosis and necroptosis in specific contexts, but it can activate apoptosis and necroptosis in other contexts (23, 24). Of high interest is to investigate *in vivo* an exact consequence of RIPK1 downregulation induced by emu-let-7-5p.

An early study assessed the effects of *E. multilocularis* infection on the expression of costimulatory molecules on peritoneal macrophages, and found that CD40 was downregulated and CD54 was slightly upregulated, but CD86 and CD80 remained unchanged (25). Conversely, the current study confirmed that CD40 was upregulated (*p* < 0.05), while CD86, CD54 and CD80 kept constant in expression in emu-let-7-5p-transfected cells compared with the control (Figure 1G). The discrepancy suggests that other molecules potentially contribute to the impairment of CD40 and CD54 expression during *E. multilocularis* infection, which needs to be further investigated in future.

Although emu-let-7-5p was *in vitro* shown to have an immunomodulatory capacity, the current study still lacks compelling evidence that supports its role in regulating immune responses in infected animals. Another limitation is that the immunoregulatory functions of emu-let-7-5p can be overestimated or lowestimated because the concentration used for transfection in this study may be lower or higher than that in the sera of mice infected by *E. multilocularis*.

## Conclusions

In summary, emu-let-7-5p was able to interfere with cytokine and surface marker expression and LPS/TLR-4 signaling pathway in the transfected macrophages, suggesting a role in pathogen-host interactions during *E. multilocularis* infection.

## Data Availability Statement

The original contributions presented in the study are included in the article/supplementary material, further inquiries can be directed to the corresponding author/s.

## Author Contributions

YZ designed the experiments. XJ, YL, and XY performed the experiments and the statistical analysis. XJ, YL, and YZ wrote the paper. YZ and XY revised the paper. All authors contributed to the article and approved the submitted version.

## Conflict of Interest

The authors declare that the research was conducted in the absence of any commercial or financial relationships that could be construed as a potential conflict of interest.
